# A multilevel analysis of the relationship between parental migration and left-behind children’s macronutrient intakes in rural China

**DOI:** 10.1017/S1368980015003341

**Published:** 2015-12-08

**Authors:** Nan Zhang, Laia Bécares, Tarani Chandola

**Affiliations:** 1 Room 3.331, The School of Nursing, Midwifery and Social Work, Jean McFarlane Building, University of Manchester, Oxford Road, Manchester M13 9PL, UK; 2 Cathie Marsh Institute for Social Research (CMIST), School of Social Sciences, University of Manchester, Manchester, UK

**Keywords:** Nutritional intakes, Malnutrition, Left-behind children, Gender, China

## Abstract

**Objective:**

China’s internal migration has left 61 million children living apart from their parent(s) in rural areas. The present study aimed to examine whether the relative contributions of macronutrients (protein, fat and carbohydrate) to total energy intake differ between children left behind by the father or mother, compared with children from intact families.

**Design:**

Drawing on a longitudinal study, the China Health and Nutrition Survey (1997–2009), multilevel modelling analyses (level 1: occasions; level 2: children; level 3: villages) were performed.

**Setting:**

Data from rural communities in nine provinces in China.

**Subjects:**

Rural children (*n* 975; 555 boys and 420 girls) from 140 villages.

**Results:**

Among boys of school age, being left behind by the father tended to reduce the relative protein intake by 0·70 % (*P*<0·01) compared with boys from intact families. Being left behind by at least the mother was more detrimental for young boys under the age of 6 years than paternal migration, reducing relative protein intake by 1·14 % (*P*<0·05). Parental migration was associated with a significant increase in young boys’ relative fat intake by 2·60 % (*P*<0·05). No significant associations were found for girls. Results suggest left-behind boys, especially in early life, are subject to a higher-fat and lower-protein diet compared with non-left-behind boys. This may put them at increased risk of being overweight or obese, or of suffering from stunted growth, when they grow up.

**Conclusions:**

Public health policies should recognise the influences of parental migration on boys, especially maternal migration, and encourage a more balanced diet for children in rural China.

China’s internal migration has resulted in about 61 million rural children under 18 years of age living apart from either one or both parents^(^ ^1^ ^)^. The majority of these children are left behind by both parents (46·5 %), followed by being left behind by the father only (36·4 %) and by the mother only (16·9 %). A number of studies in rural China have examined the associations between parental migration and children’s nutritional health in terms of anthropometric measures (including height-for-age *Z*-score, weight-for-age *Z*-score, BMI) and dietary intakes^(^ ^2^ ^,^ ^3^ ^)^, among other outcomes^(^ ^2^ ^,^ ^4^ ^–^ ^9^ ^)^, mostly suggesting a negative association between parental migration and child nutritional outcomes. However, few studies have distinguished the effect of differences in patterns of parental migration (i.e. by the gender of the parent) on children’s nutritional status^(^ ^4^ ^,^ ^7^ ^,^ ^10^ ^)^ or on children’s dietary intakes^(^ ^3^ ^)^. The gender of the child may be also important in intra-household allocation, particularly in rural China where ‘son preference’ is prevalent^(^ ^11^ ^)^.

One case–control survey conducted in Southern China showed that the average daily intake of protein-source foods including fish, eggs and meat for left-behind children (LBC) under 7 years of age was significantly lower than that of their non-left-behind peers^(^ ^2^ ^)^. However, that analysis failed to distinguish between the effects of paternal migration and maternal migration on boys and girls separately. This is an important limitation given well-documented gender differences in energy and nutrient needs^(^ ^12^ ^)^. Another study^(^ ^3^ ^)^ drawing on data from the China Health and Nutrition Survey (CHNS; 2000–2009) found that migration of both parents tended to increase the likelihood of protein and energy deficiency for children aged 6–17 years, while no significant associations were found for children left behind by one parent. LBC, especially those who were left behind by one parent, appeared to have decreased risk of fat overconsumption. However, that study did not examine whether the association between parental migration and nutritional deficiency of LBC differs by the gender of the migrant parent or by the gender of the child. This is an important limitation given the distinct gender roles of both adults and children in rural China.

The present study aimed to address these limitations by examining the impact of parental migration on rural children’s macronutrient intakes and considering the gender of the parents and the children. Analysing gender differences is important not only because of the maternal role as a traditional child-care provider within households in China, but also because of gender differences in the use of household financial resources, such as remittances. Mothers tend to purchase higher-quality foods in the absence of migrant fathers, which has important implications for the nutritional well-being of children as they develop^(^ ^13^ ^–^ ^15^ ^)^. With regard to the gender of the LBC, we expect that the effect of parental migration on nutritional intakes may differ between boys and girls partly due to gender differences in energy and nutrient needs^(^ ^12^ ^)^, but also because of gender discrimination in favour of boys in intra-household allocation in rural China^(^ ^11^ ^)^.

Another innovation of the present paper is its focus on relative macronutrient intakes expressed as the percentage of total energy intake contributed by a macronutrient including protein, fat and carbohydrate. This measurement of nutritional outcomes can, in a sense, reflect food composition and dietary quality, in contrast to previous studies using anthropometric measures. The Acceptable Macronutrient Distribution Range (AMDR) for carbohydrate, fat and protein as a general diet evaluation guide, expressed as the percentage of total energy intake, is a healthy range of intake of a particular energy source that is associated with a reduced risk of chronic disease while providing adequate amounts of essential nutrients^(^ ^16^ ^)^. Intakes outside this range raise the potential for an increased risk of chronic disease shown to be associated with long-term health and increase the risk of insufficient intakes of essential nutrients^(^ ^16^ ^)^.

In order to investigate the associations between left-behind patterns (being left behind by the father or by the mother) and children’s relative macronutrient intake status, with an emphasis on the gender differences of the parents and of the LBC, the study aimed to answer the following interrelated research questions:

1. Is parental migration associated with the relative macronutrient intakes of LBC in terms of the percentage contribution of protein, fat and carbohydrate to total energy intake?
2. Is parental migration associated with the relative macronutrient intakes of LBC being within, above or below the AMDR for protein, fat and carbohydrate?
3. Do associations examined in questions 1 and 2 differ according to the gender of the parents and the gender of the LBC?

## Methods

### Study design and participants

Data were drawn from the CHNS, an ongoing open-cohort study that employs a multistage, random-clustered sampling process to draw a sample of about 4400 households with a total of about 19 000 participants from over 200 communities or neighbourhoods in nine provinces, with the first round conducted in 1989. The CHNS covers nine provinces that vary substantially in geography, economic development, public resources and health indicators. The design, sampling and response rates are reported elsewhere^(^ ^17^ ^)^.

We used data from five waves of the CHNS, collected in 1997, 2000, 2004, 2006 and 2009. The status of being left behind was operationalised using the household roster: from 1997 onwards, if one household member in a previous round of the CHNS was not residing in the same household in the current survey, the respondent was asked for the reasons for his/her absence. In the present study, children under 18 years old whose parent(s) had left the home to seek employment elsewhere were defined as LBC. Children varying in age (between 0 and 6 years) were recruited in 1997, 2000, 2004 and 2006, and then followed up to 2009, by drawing on accelerated longitudinal designs^(^ ^18^ ^)^. Multiple cohorts at different waves included not only newborn eligible children, but also a new province that was added from 2000, and villages lost to follow-up returned in later waves^(^ ^17^ ^)^.

The institutional review committees from the University of North Carolina at Chapel Hill and the National Institute for Nutrition and Food Safety, China Centre for Disease Control and Prevention, approved the survey protocols and instruments and the process for obtaining informed consent for the survey. All participants and/or their parents/guardians provided written informed consents for their participation in the survey.

### Dietary variables

Using food models and picture aids, trained fieldworkers collected detailed individual-level 24 h recall diet data (e.g. food types, amounts, type of meal and place of consumption of all foods consumed during 24 h of the previous day) for three consecutive days^(^ ^19^ ^)^. Individual dietary intakes were obtained by asking each household member to report all foods consumed at home and away from home. Information regarding young children younger than 12 years of age was reported by caregivers who handled food preparation and feeding in the household. Household food consumption was measured on a daily basis over the same three-day period by calculating changes in household food inventory using a weighing technique. Digital diet and kitchen scales with a maximum limit of 3 kg and a minimum of 1 g were used to weigh the foods. All foods in stock within the household at the initiation of the survey, foods purchased and/or produced at home during the survey period, and food preparation waste were weighed and recorded. They were considered in the calculation of household food consumption^(^ ^19^ ^)^. At the end of the survey, all remaining foods were again weighed and recorded. Housewives and other household members were encouraged to provide additional information to determine the amounts of particular food items in dishes consumed in the household^(^ ^20^ ^)^. The amount of each dish was estimated from the household inventory and the proportion of each dish consumed was reported by each member interviewed. Therefore, individual food consumption was determined by the total amount and by the proportion that each individual ate^(^ ^21^ ^)^. Interviewers used information on household food consumption to cross-check the individual diet recall data. Where significant discrepancies were found, the household and the individual in question were revisited and asked about their food consumption to resolve these discrepancies. Individual diet data were then used to calculate the nutrient values in terms of intakes of energy, protein, fat and carbohydrate based on the food composition table for China. The dietary assessment approach of the CHNS has been shown to reduce the effects of measurement error and accurately capture usual energy intake^(^ ^22^ ^)^.

Relative macronutrient intakes were defined as the percentage of total energy intake contributed by a macronutrient including protein, fat and carbohydrate. Macronutrients can be utilised by the body for energy, with 1 g of protein and 1 g of carbohydrate yielding 16·7 kJ (4 kcal) and 1 g of fat yielding 37·7 kJ (9 kcal)^(^ ^23^ ^)^. When needed to meet the body’s energy requirements, macronutrients can, to some extent, replace each other. They are not independent of one another as energy fuel source or of the total energy requirement of the individual. For a specific level of energy intake, increasing the proportion of one macronutrient necessitates decreasing the proportion of the one or both of the other macronutrients^(^ ^16^ ^)^. Relative macronutrient intakes were compared with AMDR. The AMDR for carbohydrate is 45–65 % for children aged 1–17 years; the AMDR for fat is 30–40 % for children aged 1–3 years and 25–35 % for children aged 4–18 years; the AMDR for protein is 5–20 % for children aged 1–3 years and 10–30 % for children aged 4–18 years^(^ ^24^ ^)^.

### Left-behind status

To investigate the associations between different types of parental migration and macronutrient status of children who stay behind, we created a three-category time-variant variable to indicate whether the child was left behind: (i) by the father only; (ii) by at least the mother (combining being left behind by the mother only (*n* 31 for girls and *n* 28 for boys) and by both parents (*n* 55 for girls and *n* 34 for boys) due to small sample size in each category); or (iii) by none of the parents, referring to children from intact families.

### Covariates

We controlled for a range of sociodemographic and socio-economic variables at different levels, including age, gender, only child (whether a child is the only child within a household), child height and weight, household size, annual household income per capita and maternal education (the number of years of formal education completed). The gender of a child often affects the intra-household allocation, particularly in rural China where son preference is prevalent^(^ ^11^ ^)^. Child height and weight were included to control for individuals’ energy requirements depending on the BMR, which in turn is influenced by the height and weight of individuals^(^ ^25^ ^)^. The CHNS recorded height and weight for each individual within the household, measured by health professionals.

Controlling for whether the child is the only child within the household was done because the presence of other siblings implies competition for intra-household recourses and may reduce the amount of resources each child receives. The number of household members was also taken into account because children in a large extended family may receive less attention than a child in a nuclear family. We adjusted for maternal education because it can affect children’s health given that literacy and numeracy skills gained from education enable the mother to obtain health knowledge and therefore influence her child’s health^(^ ^26^ ^)^. An urbanisation index was created for each community (village or neighbourhood) based on twelve multidimensional components from the CHNS including population density, transportation infrastructure, access to traditional and modern markets, sanitation, housing, health infrastructure and related factors that distinguish features of urban places (detail reported elsewhere)^(^ ^27^ ^)^. This score was used to capture physical, social, cultural and economic environmental risk factors for children’s nutritional health^(^ ^28^ ^,^ ^29^ ^)^.

Regional dummies were created according to geographical and socio-economic differences: coastal (Shandong and Jiangsu, the two most economically developed provinces), north-east (Liaoning and Heilongjiang), central inland (Henan, Hubei and Hunan) and the mountainous south (Guangxi and Guizhou). This latter category, capturing the most economically deprived region in China, was set as the reference region. Regional dummies were controlled to capture unobserved geographic and cultural factors related to food consumption and food prices, which may influence dietary intakes. We also included wave dummies to capture time effects with 1997 as reference wave.

### Statistical analysis

To account for the hierarchical nature of the CHNS, where multiple occasions (level 1) are nested within children (level 2) and clustered in villages (level 3), multilevel regression models for the continuous outcomes (relative macronutrient intakes) were constructed using the statistical software package Stata version 13·1. Multinomial logit models were conducted within the software package MLwiN version 2·28 for categorical outcomes of being within, above or below the AMDR, which allowed us to compare relative macronutrient intakes with the respective AMDR and examine whether the relative macronutrient intake was within the AMDR (as reference), below the AMDR lower limit or above the AMDR upper limit. The log likelihood ratio test was used to determine the choice of model among nested models for continuous outcomes – relative macronutrient intakes. The Wald test was used to test statistical significance in multinomial logit regressions for categorical outcomes.

As AMDR are not available for children younger than 1 year old, we only included children aged 1–17 years in the final analyses. Children with non-missing values on outcome variables and key predictors were kept in the analysis, yielding a total sample of 2171 observations within 975 children (555 boys, 420 girls) clustered in 140 villages. We first examined the associations between being left behind and relative macronutrient intakes among boys and girls (model 1 and model 3). We then decomposed parental migration into two components: being left behind by the father only and being left behind by at least the mother (model 2 and model 4). Given gender differences in energy and nutrient needs^(^ ^12^ ^)^, we performed subgroup analyses based on stratification by gender for boys and girls separately. Children were separated into two age groups: pre-school children aged 1–5 years and schoolchildren aged 6–17 years, because the energy balanced-related behaviours of these two age groups tend to differ^(^ ^12^ ^)^. These analyses were based on complete cases. The interactions between left-behind types (by the father, by at least the mother and non-left-behind) and the gender of children were not statistically significant at the 5 % level (results not reported).

Due to missing data on a few key variables, including on the measures of left-behind status (*n* 1403), protein intake (*n* 1840), household income per capita (*n* 1489) and maternal education (*n* 1457), we performed multiple imputation on the data set under the assumption that the data were missing at random, whereby the missingness (i.e. whether the data are missing or not) may depend on observed data, but not on unobserved data^(^ ^30^ ^)^. In order to reduce bias, the imputation model should include a wide range of variables including all of the variables in the substantive analysis, variables that predict missingness and variables likely to be correlated with the process leading to missing data, although they may not be of interest in the substantive analysis^(^ ^31^ ^)^. These predictors could cause either downward or upward bias in our estimates. For example, we found that children from low-income families were more likely to drop out from subsequent surveys as they might experience more difficulties to participate. Poorer children may have lower nutritional intakes than their richer counterparts, which may cause a downward bias in our estimates of the effect of being left behind on children’s nutritional intakes. We also found that children with a larger family size were less likely to drop out; this could bias our estimates upwards since a larger family size may be more detrimental to children’s nutritional intakes due to restricted resource distribution. However, we were unable to differentiate the exact direction of bias that was introduced by sample attrition and/or missingness.

We included all variables used in the substantive models as well as an auxiliary variable likely to predict the attribution of child participants: whether one particular child has a grandparent or not. Multiple Imputation using Chained Equations was used to create thirty imputed data sets using the Stata 13·1 ‘mi impute chained’ command^(^ ^32^ ^)^. The number of imputed data sets was determined by the attrition rate of about 30 % in the present study, which suggests as a rule of thumb that the imputations should be at least equal to the percentage of incomplete cases in the data set^(^ ^33^ ^)^. Sensitivity analyses performed on the imputed data sets showed similar results to those from the complete cases analyses (data shown in Appendix 4), so in the following sections we present and discuss results only from the complete case analysis.

## Results

**Fig. 1:**
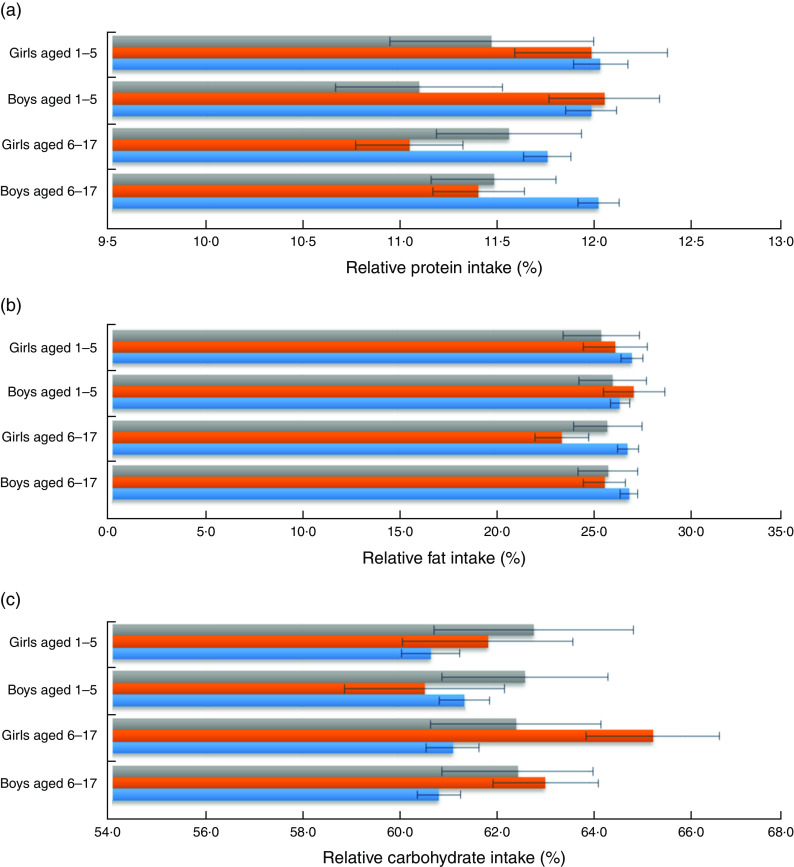
(colour online) Relative macronutrient intake (percentage contribution to total energy intake) of (a) protein, (b) fat and (c) carbohydrate of pre-school aged (1–5 years) and school-aged (6–17 years) boys and girls by left-behind patterns (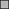, by at least mother; 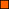, by father only; 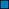, non-left-behind) in rural China. Values are means, with their standard errors represented by horizontal bars; China Health and Nutrition Survey, 1997–2009

**Fig. 2:**
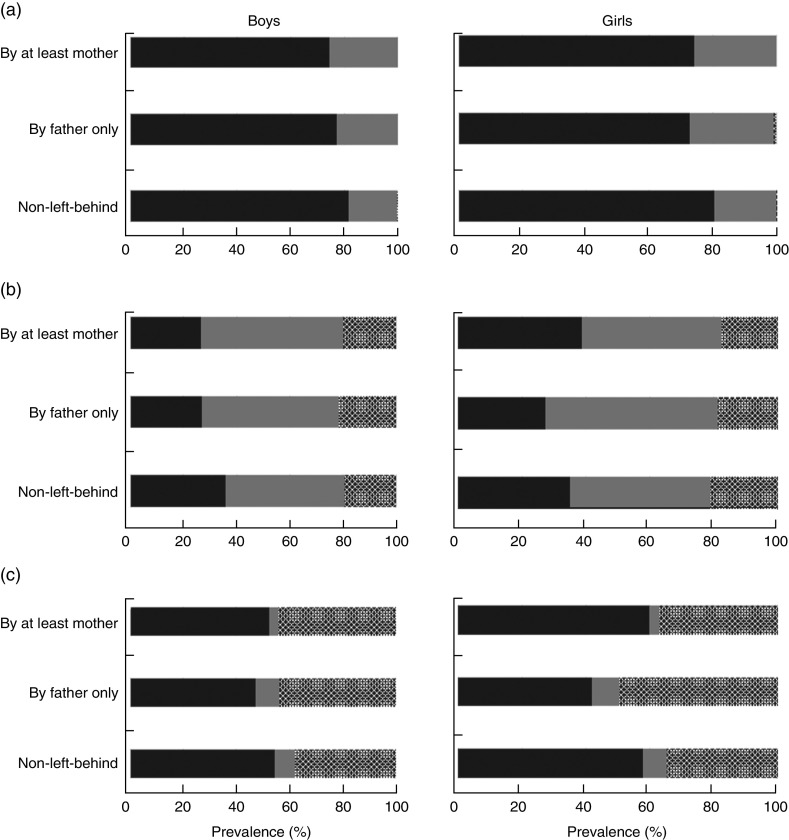
Prevalence of rural children with relative macronutrient intake (percentage contribution to total energy intake) within (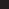), below (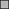) and above (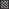) the Acceptable Macronutrient Distribution Range for (a) protein, (b) fat and (c) carbohydrate by left-behind patterns for boys and girls aged 1–17 years in rural China; China Health and Nutrition Survey, 1997–2009

Figure 1 shows relative macronutrient intakes of the CHNS children aged 1–17 years according to left-behind patterns in rural China from 1997 to 2009. The mean (and sd) (relative) macronutrient intakes for the CHNS children are presented in Appendix 1. The mean macronutrient-energy percentages for boys and girls by left-behind pattern were within the AMDR for protein, fat and carbohydrate, with relative protein and fat intakes approaching the lower limits of the AMDR (10 % and 25 %, respectively, for children aged 4–18 years) and relative carbohydrate intake approaching the AMDR upper limit (65 % for children aged 4–18 years). Young boys aged 1–5 years who were left behind by at least the mother tended to have the lowest-protein diet as compared with other young boys and girls. Young boys who were left behind by the father only had the highest-protein and highest-fat diet compared with other children. Left-behind boys and girls of school age tended to have lower percentage contributions of protein and fat to total energy intake than children from intact families. Relative carbohydrate intake, in general, appeared to be higher among LBC than among children from intact families. Figure 2 shows the proportions of children whose macronutrient intakes were within AMDR, below the AMDR lower limit and above the AMDR upper limit, according to left-behind pattern. On average, for protein intake, over 20 % of children appeared not meet the recommended protein intake and only a small proportion of children (less than 1 %) exceeded the AMDR upper limit. Over half of boys and girls who were left behind by the father only appeared to suffer from fat deficiency. Almost half of LBC appeared to consume more carbohydrate than recommended.

Table 1 presents the coefficients and se of the multilevel modelling of relative protein intakes for boys and girls of pre-school and school age after adjusting for socio-economic and demographic confounders. For pre-school boys, being left behind due to parental migration was associated with a lower relative daily protein intake of 0·46 % (model 1 for pre-school age, Table 1) compared with boys of the same age from intact families, although the association was not statistically significant (*P=*0·17). The association between being left behind with boys’ protein-energy density became statistically significant when boys were of school age (model 1 for school age, Table 1): left-behind school-aged boys, on average, appeared to consume a diet lower in protein than non-left-behind boys of the same age by 0·70 % (*P*<0·01). Being left behind by at least the mother tended to be more detrimental to young boys’ dietary protein-energy density by reducing it by 1·14 % *(P*<0·05), as compared with pre-school boys from intact families. The negative association between being left behind by at least the mother and dietary protein-energy density persisted but became less pronounced when boys grew up to school age (coefficient=−0·67, se=0·37). Being left behind by the father only was associated with a significant reduction in school-aged boys’ protein-energy density of 0·72 % (*P*<0·01), as compared with boys of the same age with both parents living at home. There were no significant associations between relative protein intake and parental migration found among pre-school girls (coefficient=−0·18, se=0·35; Table 1, model 3) and school-aged girls (coefficient=−0·36, se=0·27; Table 1, model 3).

**Table 1:** Estimates (with their standard errors) of relative protein intake (percentage contribution to total energy intake) from multilevel modelling fitted to rural children across gender and age groups from the China Health and Nutrition Survey, 1997–2009

	Pre-school aged (1–5 years)								School-aged (6–17 years)							
	Boys				Girls				Boys				Girls			
	Model 1†		Model 2†		Model 3†		Model 4†		Model 1†		Model 2†		Model 3†		Model 4†	
	%	se	%	se	%	se	%	se	%	se	%	se	%	se	%	se
Fixed effects																
Intercept	5·49	2·83	5·26	2·83	4·17	3·02	4·27	3·00	8·73	1·81	8·74	1·81	11·63	1·99	11·61	1·98
Left behind (reference: non-left-behind)	−0·46	0·32			−0·18	0·35			−0·70**	0·23			−0·36	0·27		
Log likelihood ratio test *P* value	0·17				0·61				0·003				0·19			
Left-behind types (reference: non-left-behind)																
By father only			0·02	0·39			0·21	0·42			−0·72**	0·27			−0·52	0·31
By at least mother			−1·14*	0·45			−0·71	0·50			−0·67	0·37			0·01	0·43
Log likelihood ratio test *P* value			0·04				0·27				0·01				0·24	
Covariates																
Age‡ (years)	−0·25	0·18	−0·25	0·18	−0·42*	0·20	−0·41*	0·20	−0·05	0·08	0·05	0·08	0·12	0·09	0·12	0·09
Only child (reference: have siblings within the same household)	0·28	0·26	0·31	0·26	0·30	0·31	0·34	0·31	0·26	0·21	0·26	0·21	−0·41	0·26	−0·42	0·26
Household income per capita/1000 (RMB)	0·02	0·03	0·02	0·03	0·001	0·03	0·005	0·03	0·03*	0·01	0·03*	0·01	0·02	0·02	0·02	0·02
Maternal education (years)	0·11*	0·04	0·11**	0·04	0·08	0·04	0·08	0·04	0·09**	0·03	0·09**	0·03	0·11**	0·04	0·11**	0·04
Household size	0·18*	0·09	0·20*	0·08	0·08	0·09	0·08	0·09	0·05	0·08	0·05	0·08	−0·10	0·09	−0·11	0·09
Urbanisation score	0·03***	0·01	0·03***	0·01	0·04***	0·01	0·04***	0·01	0·01	0·01	0·01	0·01	0·02**	0·01	0·02*	0·01
−2 Log likelihood	−1303·55		−1301·30		−922·75		−921·58		−1558·91		−1558·90		−1183·28		−1182·72	

*P<0·05, **P<0·01, ***P<0·001.

† Additionally adjusted for children’s height, children’s weight, regional dummies and wave dummies, which were not reported here.

‡ Age was centred on 4 years for pre-school aged children and 10 years for school-aged children.

Table 2 presents the multilevel modelling results of relative contribution of fat to total energy intake for boys and girls of pre-school and school age. For pre-school boys, being left behind was associated with a significant increase in the percentage of dietary energy from fat sources by 2·60 % (*P*<0·05), on average, as compared with boys of the same age from intact families (model 1 for pre-school age, Table 2). When decomposing the status of being left behind into two components as shown in model 2 for school age from Table 2, results suggested that young boys who were left behind by the father only tended to have a higher relative fat intake (coefficient=2·91, se=1·50) compared with boys of the same age who were left behind by at least the mother (coefficient=2·13, se=1·76), on average. Both groups of left-behind children had higher relative intake than non-left-behind pre-school boys from intact families. The positive association of being left behind on relative fat intake for boys became less pronounced and lost statistical significance when boys entered school age (model 1 and model 2 for school age, Table 2).

**Table 2:** Estimates (and their standard errors) of relative fat intake (percentage contribution to total energy intake) from multilevel modelling fitted to rural children across gender and age groups from the China Health and Nutrition Survey, 1997–2009

	Pre-school aged (1–5 years)								School-aged (6–17 years)							
	Boys				Girls				Boys				Girls			
	Model 1†		Model 2†		Model 3†		Model 4†		Model 1†		Model 2†		Model 3†		Model 4†	
	%	se	%	se	%	se	%	se	%	se	%	se	%	se	%	se
Fixed effects																
Intercept	5·21	10·21	5·03	10·94	7·87	12·44	8·08	12·44	10·54	7·69	10·74	7·70	4·79	9·00	4·87	8·97
Left behind (reference: non-left-behind)	2·60*	1·23			1·42	1·45			0·35	0·98			0·22	1·21		
Log likelihood ratio test *P* value	0·04				0·33				0·72				0·86			
Left-behind types (reference: non-left-behind)																
By father only			2·91	1·50			2·16	1·75			−0·02	1·15			−1·19	1·38
By at least mother			2·13	1·76			0·32	2·06			1·13	1·56			3·44	1·94
Log likelihood ratio test *P* value			0·10				0·47				0·77				0·11	
Covariates																
Age‡ (years)	−0·22	0·70	−0·22	0·70	−0·43	0·82	−0·42	0·82	−0·90**	0·34	−0·89**	0·34	−1·01*	0·41	−1·05*	0·41
Only child (reference: have siblings within the same household)	2·95**	1·02	2·97**	1·02	−0·46	1·27	−0·40	1·27	−0·23	0·89	−0·22	0·89	2·84*	1·19	2·75*	1·19
Household income per capita/1000 (RMB)	0·18	0·12	0·18	0·11	0·09	0·10	0·09	0·10	0·07	0·06	0·07	0·06	0·15	0·10	0·16	0·10
Maternal education (years)	0·33*	0·16	0·34*	0·16	0·42*	0·18	0·41*	0·18	0·07	0·14	0·06	0·14	0·19	0·18	0·18	0·18
Household size	−0·86*	0·34	−0·84*	0·34	−0·47	0·38	−0·47	0·38	−0·55	0·35	−0·57	0·35	−0·68	0·43	−0·74	0·42
Urbanisation score	0·17***	0·04	0·17***	0·04	0·15***	0·05	0·15***	0·05	0·20***	0·04	0·20***	0·04	0·12*	0·05	0·12*	0·05
−2 Log likelihood	−2049·22		−2049·16		−1494·63		−1494·34		−2562·60		−2562·40		−1966·95		−1964·77	

*P<0·05, **P<0·01, ***P<0·001.

† Additionally adjusted for children’s height, children’s weight, regional dummies and wave dummies, which were not reported here.

‡ Age was centred on 4 years for pre-school aged children and 10 years for school-aged children.

Table 3 summarises the analyses on the association between the left-behind variables and the multinomial outcomes of being below or above the recommended AMDR limits. Only the outcomes of being above or below the recommended AMDR limits for fat and carbohydrate were examined and reported. For the outcome for protein, the sample size of children whose protein intake exceeded the AMDR upper limit was insufficient to achieve modelling convergence. Left-behind boys were more likely to exceed the upper limit of recommended fat intake compared with boys from intact families (*P*<0·01; Table 3). There were no statistically significant associations between left-behind status and relative fat intake among pre-school and school-aged girls (Tables 2 and 3), or between left-behind status and relative carbohydrate intake among boys and girls (results shown in Appendices 2 and 3).

**Table 3:** Selected parameter estimates (and their standard errors) from a multinomial logit model (using within AMDR as the reference) for two scenarios of relative fat intake (percentage contribution to total energy intake), including below the AMDR lower limit and above the AMDR upper limit, for boys and girls aged 1–17 years in rural China, with adjustment for clustering at individual and village levels, from the China Health and Nutrition Survey, 1997–2009

	Boys								Girls							
	Model 1†				Model 2†				Model 3†				Model 4†			
	Below AMDR lower limit		Above AMDR upper limit		Below AMDR lower limit		Above AMDR upper limit		Below AMDR lower limit		Above AMDR upper limit		Below AMDR lower limit		Above AMDR upper limit	
	%	se	%	se	%	se	%	se	%	se	%	se	%	se	%	se
Fixed effects																
Left behind (reference: non-left-behind)	0·32	0·17	0·56**	0·20					−0·11	0·19	0·17	0·24				
Wald test *P* value	0·053		0·005						0·55		0·47					
Left-behind types (reference: non-left-behind)																
By father only					0·32	0·20	0·50*	0·24					0·13	0·22	0·33	0·28
By at least mother					0·33	0·25	0·69*	0·30					−0·54	0·30	−0·09	0·36
Wald test *P* value					0·15		0·013						0·13		0·44	

AMDR, Acceptable Macronutrient Distribution Range. *P<0·05, **P<0·01.

† Additionally adjusted for child age, only child, children’s height, children’s weight, maternal education, household income per capita, urbanisation score, household size, regional dummies and wave dummies, which were not reported here.

## Discussion

The present study aimed to examine the association between parental migration and children’s relative macronutrient intakes (i.e. percentage of total energy intake from protein, fat, carbohydrate) in rural China and to explore gender differences of both parents and children in these associations. Being left behind appeared to decrease boys’ (but not girls’) relative protein intake and to increase their relative fat intake. Young boys who were left behind, especially by at least the mother, tended to have a lower-protein but higher-fat diet as compared with boys from intact families. No significant associations between left-behind status and relative macronutrient intakes were observed for girls.

Previous longitudinal evidence^(^ ^3^ ^)^ has suggested that both parents’ migration tends to increase the likelihood of protein and energy intake deficiency for children aged 6–17 years, while no significant associations were reported for children who are left behind only by one parent compared with children who are not left behind. LBC, especially those who were left behind by one parent, appear to have decreased risk of fat overnutrition^(^ ^3^ ^)^. However, in that study the nutrient deficiency was poorly defined and potentially overestimated, as the authors used the recommended nutrient intake, which represents an optimal intake of the nutrient that exceeds the requirement of 97–98 % of the population, instead of using an estimated averaged intake which meets the requirements of 50 % of healthy individuals^(^ ^34^ ^)^ and therefore provides a more conservative estimate of nutrient deficiency among LBC. Moreover, that previous analysis failed to distinguish gender differences in the associations between parental migration patterns and children’s nutritional intakes. After examining gender differences in the present study, we find that parental migration tends to decrease the proportion of energy available from protein, but not from non-protein sources including fat and carbohydrate, among rural boys in China. Protein intakes, especially from animal products, are essential for optimal growth in children and adolescents^(^ ^35^ ^)^, but are still limited for poor families in the developing world^(^ ^36^ ^)^. Left-behind boys, especially those of school age who have relative lower protein intakes as compared with boys from intact families in rural China, are vulnerable to stunted growth. Foods rich in carbohydrate are an essential part of the traditional Chinese diet^(^ ^37^ ^)^. Evidence suggests that nutritional intakes of Chinese households have not increased *pari passu* (equally) with their household income growth due to rising prices of protein-source foods, which can offset the positive income effects on the diets of children^(^ ^38^ ^)^. This may suggest that protein (especially animal protein) intake is more sensitive to increased household economic status than other sources of energy, including carbohydrates.

We found that young boys who were left behind appeared to have a higher-fat diet than boys from intact families. Left-behind boys were also more likely to exceed the recommended fat intake as compared with boys from intact families. This may cause certain nutritional problems for left-behind boys in rural China. High intakes of fat during childhood, especially saturated fat, may potentially contribute to future overweight or obese status and chronic heart disease, although the evidence for this is tenuous^(^ ^16^ ^)^. Studies show that in the last two decades Chinese children in rural areas have been undergoing a dramatic nutritional transition from a traditional low-fat and high-carbohydrate diet to a high-fat diet, especially among children from relatively affluent families^(^ ^39^ ^)^. Our findings suggest that both increased socio-economic status at the household level and increased urbanisation at the neighbourhood level appear to increase rural children’s relative protein and fat proportion, but reduce the percentage contribution of carbohydrate to total energy intake. The prevalence of overweight/obesity has increased among rural children in China^(^ ^40^ ^,^ ^41^ ^)^, which may lead to a future increase in non-communicable disease in this population^(^ ^42^ ^)^.

LBC in rural China are often left in the care of close relatives, such as grandparents^(^ ^1^ ^)^. The nutritional knowledge and food preferences of LBC’s caregivers can contribute to children’s eating habits. Evidence suggests that non-parent caregivers (mainly grandparents) of LBC tend to have relatively poorer nutritional knowledge and behaviours than parent caregivers^(^ ^43^ ^,^ ^44^ ^)^. Although malnutrition occurs primarily due to inadequate dietary intake, which is rooted in disadvantaged economic status, evidence shows that the high rates of malnutrition among children are due largely to a lack of knowledge with respect to healthy dietary intake rather than food shortage^(^ ^43^ ^,^ ^45^ ^)^.

The positive association between parental migration and young boys’ fat intake should be interpreted with caution: our descriptive analysis among left-behind boys showed that over half of them were at risk of fat intake deficiency, while only one-fifth of them seemed to overconsume fat. Young children have a higher fat oxidation than adults and low-fat diets can lead to reduced intake of certain micronutrients, including fat-soluble vitamins^(^ ^16^ ^)^. This suggests that increased fat composition due to parental migration can be beneficial to the majority of left-behind young boys. However, whether and to what extent parental migration benefits rural children’s fat intakes still needs further exploration.

Without direct comparisons between boys and girls, we were able to observe some gender differences in the associations between parental migration and relative macronutrient status. The magnitude of the association of being left behind with protein-energy percentage for boys appeared to be larger than that of girls, and the different left-behind patterns (by the father only and by at least the mother) did not appear to affect girls’ relative macronutrient intakes, as it did boys’. This may suggest that being left behind can be more detrimental for boys than for girls. One possible explanation may be due to socio-cultural factors; for example, migrant parents of boys tend to save up for sons’ adult lives rather than sending financial remittances immediately in the context of rural China^(^ ^46^ ^)^. This may imply that less financial resources are spent on left-behind boys that could be important for their growth and development. However, there is evidence that girls are more likely than boys to be disadvantaged in nutrient intakes due to the ‘son preference’ norm in rural China^(^ ^3^ ^)^. These findings, however, should be interpreted with caution due to relatively small samples who were left behind by at least the mother for boys (*n* 86) and girls (*n* 62).

Our findings suggest that maternal migration can be more detrimental to young children’s and especially young boys’ relative protein intake than paternal migration. There are some possible explanations for these patterns. First, migrant women tend to earn less than migrant men in China^(^ ^47^ ^)^, which may affect the amount of remittances sent back to LBC. Second, women in most cultures serve as the direct caregivers of children^(^ ^48^ ^)^, including in China^(^ ^49^ ^)^. Grandparents, the primary non-parent carers of LBC in rural China, may have less healthy dietary behaviours and nutritional knowledge than LBC’s parents^(^ ^43^ ^,^ ^50^ ^)^. Third, gender differences have been reported in the use of household economic resources such as remittances. For example, mothers tend to purchase higher-quality foods in the absence of migrant fathers^(^ ^13^ ^–^ ^15^ ^)^. For older children (boys in particular) of school age from 6 to 17 years, being left behind by the father only appears to be more detrimental than being left behind by at least the mother. One possible explanation is that other factors such as food environment at school and food preferences, which were not adjusted for in our study, can play a role in older children’s dietary intakes^(^ ^51^ ^)^. This is an important topic that requires future research.

Several methodological limitations warrant cautious interpretation of our findings. First, being left behind was identified based on the status of parental migration on particular time points with a minimum of a two-year interval (1997, 2000, 2004, 2006 and 2009). Parental migration status could have changed between these time points. This may have underestimated the numbers of LBC. The numbers left behind by at least the mother were relatively small, leading to potentially large se. Second, certain omitted time-varying confounders were not adjusted for in the present study, including remittances from migrant parent(s)^(^ ^52^ ^)^ and the caregiving arrangements for LBC, especially caregivers’ nutritional knowledge^(^ ^53^ ^,^ ^54^ ^)^. We controlled for maternal education because literacy and numeracy skills gained from education enable a mother as a primary caregiver for children to obtain health-related knowledge and therefore influence her children’s health^(^ ^26^ ^)^. Although the mother may take up migration work, she could still play an important role in LBC’s health and nutrition through regular long-distance communication^(^ ^55^ ^)^. Further, it has been suggested that the nutrition and health of mothers during pregnancy and lactation are associated with the health of their children throughout their lives^(^ ^56^ ^)^. However, we were unable to control for maternal nutritional health in the early years of their children’s lives in our study. Finally, measurement errors may occur when eliciting dietary data from children. The mother or caregiver reported the data on behalf of young children less than 12 years of age. Diet information was based on individual recall over a 24 h period, although steps were taken by interviewers to obtain accurate information^(^ ^22^ ^)^.

Another caveat of our study is that we did not consider the energy-related activities that partly determine children’s energy and nutrient needs^(^ ^57^ ^)^. Information about physical activities of children is available only from 2004 onwards in the CHNS, and data on pre-schoolers are substantially missing. Previous studies using the CHNS data did not find a significant association between maternal employment and the physical activities of children aged 6–18 years in China^(^ ^58^ ^)^. Moreover, LBC, especially girls, tend to engage in increased household workloads^(^ ^59^ ^)^, especially when being cared for by elderly grandparents^(^ ^50^ ^)^. This suggests that, on average, LBC may need greater energy intake compared with their peers from intact families. Finally, one key limitation in the CHNS, as in any longitudinal data set, is sample attrition. Although imputed analyses did not make much difference compared with completed cases analyses (Appendix 4), sample attrition could still lead to biased results when the missing at random assumption is violated.

Although we are unable to make definitive statements and causal inferences using the CHNS data, our findings suggest that left-behind boys, especially in early life, tend to have a higher-fat but lower-protein diet compared with their non-left-behind peers, which may put them at increased risk of becoming overweight or obese, as well as suffering from possible stunted growth, when they grow up. These potential nutritional problems for LBC, especially for young boys, have important policy implications. Public health policies should recognise the importance of parental migration, especially maternal migration, to ensure a more balanced diet for children in rural China.

## Appendix 1

**Table:** Mean (and standard deviation) macronutrient intakes of boys and girls by left-behind patterns in rural China from the China Health and Nutrition Survey, 1997–2009

			Macronutrient intake								Relative macronutrient intake (percentage contribution to total energy intake)					
			Energy (kcal)†		Protein (g)		Fat (g)		Carbohydrate (g)		Protein (%)		Fat (%)		Carbohydrate (%)	
	*n*	%	Mean	sd	Mean	sd	Mean	sd	Mean	sd	Mean	sd	Mean	sd	Mean	sd
Boys																
Age group																
Aged 1–5 years	551	44·26	1097·01	494·35	32·48	16·15	33·15	21·65	167·20	84·48	11·95	2·76	26·65	10·95	61·39	11·35
Aged 6–17 years	694	55·74	1726·80	612·67	51·26	20·72	52·36	31·34	262·58	103·21	11·93	2·51	26·84	10·58	61·24	10·54
Left-behind patterns																
Non-left-behind	1015	81·53	1436·22	646·46	42·94	21·36	43·82	29·62	217·46	104·95	12·03	2·67	26·87	10·69	61·09	10·87
By father only	144	11·57	1508·07	638·97	43·62	19·32	44·37	26·91	233·86	113·45	11·67	2·23	26·38	11·02	62·11	11·21
By at least mother	86	6·91	1487·48	635·48	41·85	19·65	43·43	26·28	232·18	110·92	11·32	2·48	26·08	10·88	62·57	10·75
Girls																
Age group																
Aged 1–5 years	403	43·52	1045·56	406·03	31·34	14·09	32·21	21·00	157·52	65·59	12·02	2·62	27·03	10·84	60·96	11·13
Aged 6–17 years	523	56·48	1616·56	1378·82	45·19	16·71	53·08	143·53	239·30	88·25	11·66	2·49	26·47	11·44	61·81	11·41
Left-behind patterns																
Non-left-behind	747	80·67	1366·13	1206·13	39·12	17·11	45·32	121·03	200·28	87·27	11·90	2·56	27·11	11·11	60·94	11·19
By father only	117	12·63	1402·62	558·65	39·65	17·28	38·50	24·04	224·29	105·02	11·41	2·53	24·61	11·83	64·03	11·96
By at least mother	62	6·70	1326·04	428·39	38·70	16·24	38·46	21·30	206·20	69·80	11·54	2·48	25·80	10·45	62·63	10·61

† 1 kcal=4·184 kJ.

## Appendix 2

**Table:** Estimates (and their standard errors) of relative carbohydrate intake (percentage contribution to total energy intake) from multilevel modelling fitted to rural children across gender and age groups from the China Health and Nutrition Survey, 1997–2009

	Pre-school aged (1–5 years)								School-aged (6–17 years)							
	Boys				Girls				Boys				Girls			
	Model 1†		Model 2†		Model 3†		Model 4†		Model 1†		Model 2†		Model 3†		Model 4†	
	%	se	%	se	%	se	%	se	%	se	%	se	%	se	%	se
Fixed effects																
Intercept	89·43	11·25	89·85	11·25	86·96	12·38	86·66	12·37	79·00	7·51	78·74	7·5	83·50	8·81	83·38	8·76
Left behind (reference: non-left-behind)	−2·10	1·27			−1·18	1·44			0·57	0·96			0·19	1·18		
Log likelihood ratio test *P* value	0·10				0·42				0·56				0·87			
Left-behind types (reference: non-left-behind)																
By father only			−2·90	1·54			−2·15	1·74			1·04	1·12			1·73	1·35
By at least mother			−0·92	1·81			0·29	2·05			−0·43	1·52			−3·32	1·89
Log likelihood ratio test *P* value			0·17				0·43				0·59				0·07	
Covariates																
Age‡ (years)	0·45	0·72	0·45	0·72	0·76	0·81	0·75	0·81	0·92**	0·33	0·91**	0·33	0·89*	0·40	0·93*	0·40
Only child (reference: have siblings within the same household)	−3·20**	1·05	−3·25**	1·05	0·31	1·26	0·24	1·26	−0·01	0·87	−0·02	0·87	−2·47*	1·17	−2·37**	1·16
Household income per capita/1000 (RMB)	−0·19	0·12	−0·20	0·12	−0·10	0·10	−0·10	0·10	−0·10	0·06	−0·10	0·06	−0·16	0·10	−0·17	0·10
Maternal education (years)	−0·44**	0·17	−0·45**	0·17	−0·48**	0·18	−0·48**	0·18	−0·17	0·13	−0·16	0·13	−0·30	0·18	−0·30	0·18
Household size	0·67	0·35	0·63	0·35	0·38	0·38	0·37	0·38	0·49	0·34	0·52	0·34	0·79	0·42	0·85*	0·41
Urbanisation score	−0·21***	0·04	−0·20***	0·04	−0·19***	0·05	−0·19***	0·05	−0·20***	0·04	−0·20***	0·04	−0·14**	0·05	−0·14**	0·05
−2 Log likelihood	−2063·72		−2063·31		−1492·94		−1492·44		−2545·20		−2544·85		−1954·94		−1952·22	

**P*<0·05, ***P*<0·01, ****P*<0·001.

† Additionally adjusted for children’s height, children’s weight, regional dummies and wave dummies, which were not reported here.

‡ Age was centred on 4 years for pre-school aged children and 10 years for school-aged children.

## Appendix 3

**Table:** Selected parameter estimates (and their standard errors) from a multinomial logit model (using within AMDR as the reference) for two scenarios of relative carbohydrate intake (percentage contribution to total energy intake), including below the AMDR lower limit and above the AMDR upper limit, for boys and girls aged 1–17 years in rural China, with adjustment for clustering at individual and village levels, from the China Health and Nutrition Survey, 1997–2009

	Boys								Girls							
	Model 1†				Model 2†				Model 3†				Model 4†			
	Below AMDR lower limit		Above AMDR upper limit		Below AMDR lower limit		Above AMDR upper limit		Below AMDR lower limit		Above AMDR upper limit		Below AMDR lower limit		Above AMDR upper limit	
	%	se	%	se	%	se	%	se	%	se	%	se	%	se	%	se
Fixed effects																
Left behind (reference: non-left-behind)	0·22	0·31	0·04	0·17					0·13	0·37	0·05	0·20				
Wald test *P* value	0·48		0·81						0·72		0·82					
Left-behind types (reference: non-left-behind)																
By father only					0·48	0·34	0·12	0·20					0·54	0·41	0·30	0·23
By at least mother					−0·50	0·60	−0·09	0·26					−0·85	0·76	−0·40	0·31
Wald test *P* value					0·22		0·75						0·18		0·13	

AMDR, Acceptable Macronutrient Distribution Range.

† Additionally adjusted for child age, only child, children’s height, children’s weight, maternal education, household income per capita, urbanisation score, household size, regional dummies and wave dummies, which were not reported here.

## Appendix 4

**Table:** Estimates (and their standard errors) of relative protein and fat intakes (percentage contributions to total energy intake) from multilevel modelling based on imputed data sets (n 30) for rural children from the China Health and Nutrition Survey, 1997–2009

	Relative protein intake (%)								Relative fat intake (%)							
	Boys†				Girls†				Boys†				Girls†			
	Aged 1–5 years (*n* 798)		Aged 6–17 years (*n* 795)		Aged 1–5 years (*n* 610)		Aged 6–17 years (*n* 591)		Aged 1–5 years (*n* 798)		Aged 6–17 years (*n* 795)		Aged 1–5 years (*n* 610)		Aged 6–17 years (*n* 591)	
	%	se	%	se	%	se	%	se	%	se	%	se	%	se	%	se
Left behind (reference: non-left-behind)	−0·36	0·28	−0·72	0·22	−0·05	0·34	−0·39	0·25	1·35	1·12	−0·09	0·91	2·05	1·32	−0·35	1·12
*P* value	0·20		0·001***		0·89		0·12		0·23		0·92		0·12		0·76	

****P*<0·001.

† Additional variations that predicted missingness including children’s height, children’s weight, children’s age, only child, maternal education, household size, urbanisation score, regional dummies, wave dummies and whether a child has a grandparent, which were not reported here.
